# Allowing more time to ILCOR Step A of neonatal resuscitation leads to better residents’ task completion in simulated scenarios. A problem of time pressure?

**DOI:** 10.1186/s12887-020-02217-3

**Published:** 2020-07-03

**Authors:** Claire Boithias, Laure Jule, Stephanie Le Foulgoc, Gilles Jourdain, Dan Benhamou

**Affiliations:** 1 Service de Réanimation Pédiatrique et Médecine Néonatale, Hôpital Bicêtre, Hôpitaux Universitaires Paris Sud (APHP) et Centre de simulation LabForSIMS, Université Paris Saclay, Le Kremlin-Bicêtre, France; 2 Service de Réanimation Néonatale, Centre Hospitalier Sud Francilien, Corbeil, France; 3 Centre de simulation LabForSIMS, Université Paris Saclay, Le Kremlin-Bicêtre, France; 4 SMUR 92 Pédiatrique et Réanimation Néonatale, Hôpital Antoine Béclère, Hôpitaux Universitaires Paris Sud (APHP) et Centre de simulation LabForSIMS, Université Paris Saclay, Le Kremlin-Bicêtre, 157 rue de la porte de Trivaux, 92140 Clamart, France; 5 Département d’Anesthésie Réanimation, Hôpital Bicêtre, Hôpitaux Universitaires Paris Sud (APHP) et Centre de simulation LabForSIMS, Université Paris Saclay, Le Kremlin-Bicêtre, France

**Keywords:** Simulation; Neonatal resuscitation; Delivery room; Time pressure; ILCOR; LabForSIMS

## Abstract

**Background:**

Roughly 10% of newborns need help to complete the transition of birth. For these infants, international guidelines recommend supporting them using a 4-step procedure (A to D). Step A is an assessment time, which includes eight tasks and finishes by starting the positive pressure ventilation (PPV), if necessary (step B). The guidelines changed in 2015 and the allotted time was raised from 30 to 60 seconds for step A completion. This study aimed to assess if the reduced time constraint in step A could have an impact on 1st-year pediatric residents' performance to complete step A and if could lead to later initiation of step A.

**Methods:**

Using video recordings of standardized neonatal scenarios over 6 years (3 before the change and 3 after), we assessed the ability of 1st-year pediatric residents of the Paris region to complete step A and initiate PPV in the allotted time in each period. Among the sessions, including at least five scenarios we evaluated all the PPV required scenarios executed for the first time by a dyad of 1st-year pediatric residents.

**Results:**

Among 52 sessions, we included 104 scenarios (25 sessions and 50 scenarios before the change and 27 sessions and 54 scenarios after). PPV started roughly at 1-minute resuscitation in both periods, but completion of the tasks before PPV-start was significant. Only 12% of the dyad of residents executed the eight tasks before PPV initiation in the first period versus 54% in the second period (*p <* 0.0001). Additionally, the completion of the eight tasks of step A was significantly better during the second period (6 [6-7] vs. 8 [7-8] *p <* 0.001).

**Conclusions:**

These results could suggest that a reduced time constraint for step A imposed by the new Guidelines was associated with better performance.

## Background

Roughly 10% of newborns do not adapt correctly and need speedy and adequate resuscitation, as indicated by the International Liaison Committee on Resuscitation (ILCOR). The ILCOR recommends that neonatal resuscitation be performed in a stepwise manner. Four steps are defined (A, B, C, D). In short, step A is an assessment of the newborn clinical status, step B initiates positive pressure ventilation (PPV), while in step C, chest compressions are started and in step D epinephrine is injected. ILCOR publishes recommendations for newborn resuscitation and updates them every five years. Medical societies such as the European Resuscitation Council publish guidelines according to these recommendations. Guidelines published in 2010 [1] were replaced in 2015 [2], changing the times required for completing step A and initiating step B. In the two versions, step B needs to be started only after the entire step A completion. However, the duration of step A is different. Initially, step A was short with a fixed 30-second duration. In the 2015 version, step A can last a maximum of 60 seconds.

Before moving forward, we have to define “time pressure” and “time constraint”. Time constraint has been defined as the difference between the amount of available time and the amount of time required to resolve a decision task [3, 4]. We can set time pressure as a subjective experience of time constraint within the context of negative consequences [5–7].

This single modification should theoretically lead to later initiation of PPV. On the other hand, however, the reduced time constraints could have an impact on step A completion. This study aimed to assess how, entirely and quickly, junior-level pediatric residents performed step A and when they initiated PPV during simulated neonatal resuscitation scenarios, before and after the new guidelines. We hypothesized that less time constraint (difference between available time and required time to perform an action) would decrease time pressure (the subjective impression of time constraint) and might have an impact on residents’ performance in completion of step A, and at the same time, we wanted to know if it leads to later initiation of PPV.

## Methods

The study was performed in the Paris Sud University simulation center (LabForSIMS) at the University Hospital Bicêtre, France, in a dedicated laboratory with a realistic, simulated delivery room with real medical equipment. The SimNewB™ simulator mannequin (Laerdal, Stavanger, Norway) was used for the study. PPV was provided by a neonatal mask and a T-piece ventilator (Neopuff™ Infant Resuscitator, Fisher & Paykel, Auckland, New Zealand). Sessions were video recorded by two cameras and sound amplified by ambient sound recorders and individual microphones worn by each trainee. According to the French national regulation, this type of study does not require any IRB approval or waiver, since it is not performed on patients' data. However, all trainees gave informed consent to session recordings and their use for scientific purposes.

The training sessions were part of the mandatory teaching of a newborn’s resuscitation for first-year pediatric residents of the Paris region and included a classroom-style course for one day followed by simulation-based training for a half-day (4 hours). The training sessions were organized during each academic year from January to June. The organization of the course was standardized and did not vary during the study period. A group of 9 to 11 students was enrolled in each session, overseen by 3 to 4 instructors who were both experienced in simulation and specialized in neonatal resuscitation. Instructors’ roles were allocated before each scenario: either as debriefer watching the scenario with the observers in the debriefing room, as a computer manager in the control room, or as a scenario facilitator (most often playing the role of the midwife). In case of an available fourth trainer, this trainer would be a co-debriefer and also watched the scenario in the control room.

A 20-minute briefing covering general teaching about European guidelines and the principles of simulation-based training preceded the sessions. The slideshow used during the briefing was overall the same during the whole study period, the only modifications in period 2 concerned one slide showing the duration of step A and another one showing that routine intubation should not be performed for tracheal suction before PPV start for non-vigorous infants born with meconium-stained amniotic fluid. Each session comprised of 5 or 6 scenarios. Each scenario began with a short oral presentation of the medical situation. The scenarios were designed to evaluate a specific educational objective, and all scenarios covered at least step A. A pair of trainees participated in each scenario, and videos were broadcast live in the debriefing room in which the other participants observed. A structured debriefing by trained instructors took place immediately after each scenario.

The same educational progression with specific learning objectives was maintained in all sessions (see Table in Supplemental Digital Content 1, which shows the structure of our sessions). In the first scenario, the baby was born tonic in clear amniotic fluid, and PPV was not required. In the second scenario, the baby was born *non-vigorous* in clear amniotic fluid, though it was always a relatively easy scenario requiring only mask ventilation. In the third scenario, the baby was born non-tonic in an amniotic meconium fluid. In the fourth and fifth scenarios, the baby was born *non-vigorous* (requiring PPV) but with increased medical complexity. The sixth one was optional and was not included in the study. To achieve a higher level of reproducibility in running a scenario for the different groups of residents, all the scenarios were preprogrammed.

We included the scenarios which had been performed by first-year pediatric residents during six consecutive academic years (2013-2018). We separated the training sessions into two periods, i.e., before (2013, 2014, 2015) and after (2016, 2017, 2018), the new guidelines were published in October 2015. As the course is organized between January and June for each academic year, 2015 scenarios were included in period 1.

To be able to assess resident performance in the same conditions in both periods, we included only the 2^nd^ and 4^th^ scenarios of each session (see Tables in Supplemental Digital Content 2 and 3, which show scenarios used for the study). We excluded all of the 1^st^ scenarios because it did not require PPV use and all the 5^th^ scenarios as at least one of the two participants had often previously participated in another scenario in the same session. Additionally, we excluded all the 3^rd^ scenarios because step B was different for non-tonic infants born with meconium-stained amniotic fluid in period 1 with routine intubation for tracheal suction before PPV. Even if step B has been similar -- whatever the color of amniotic fluid in the current guidelines -- we also excluded the 3^rd^ scenario in period 2 to limit the bias related to the change.

Because the mannequin could not move from the resuscitation table, the mannequin was covered on the table before birth. The scenario began when the scenario facilitator who played the role of the midwife came into the resuscitation room and removed the cover. The start time of PPV was defined when the resident occluded the T piece for the first time.

Before the study, we reported in a checklist the nine items required during step A for a term newborn according to national and international guidelines. Although the 2010 guidelines did not clearly recommend a method to assess heart rate (HR), we have been teaching the residents since 2012 to evaluate HR by ECG monitoring in our learning center, given the inaccuracy of clinical methods [8, 9] and the superiority of the ECG versus oximetry [10]. Since there was difficulty detecting differences between the activities of "stimulating the baby" and "drying the baby," we grouped these items such that eight tasks were evaluated for each scenario for both periods (Table 1).

**Table 1:** Checklist. Caption: Checklist of initial assessment tasks of Step A as defined by the European and the French guidelines, completed in by video reviewers for the study

Date of the session (fill out only after reviewing)			
Scenario number:			
Name of the scenario:			
Reviewer‘s name:			
**Tasks**	**Before PPV start**	**After PPV start**	**Not performed**
Apgar clock (Correct if it is the 1^st^ task executed)			
Cap (Correct if the task was finished before PPV start)			
Drying Stimulating (Correct if the task was finished before PPV start)			
Oro pharyngeal suction (Correct if the task was finished before PPV start)			
Nose suction (Correct if the task was finished before PPV start)			
Temperature probe (Correct if the task was finished before PPV start)			
HR assessment (3-lead ECG) (Correct if the task was finished before PPV start)			
Oximetry sensor (Correct as long as the task was beginning at PPV start)			
PPV start	Time : sec		

For the sake of this study, two instructors reviewed all available videos of the scenarios. They did not know the date of the sessions, only a random assigned number. The instructors assessed each task of step A, and the time of PPV-start. The instructors filled out the checklist described (in table 1). In case of discordance between reviewers, the video was reviewed jointly to reach a consensus. The duration of scenarios and debriefings were evaluated and kept for further analysis.

Results were analyzed using the STATA statistical software (StataCorp LLC, Texas 77845-4512, USA). Gaussian distribution of data was evaluated by Shapiro-Wilk test. The Welsh’s t-test and Pearson’s chi-squared test were used to compare groups when appropriate. For multivariate analysis, linear regression model was used. All tests were two-sided, and a *p* value < 0.05 was considered significant.

## Results

**Fig. 1:**
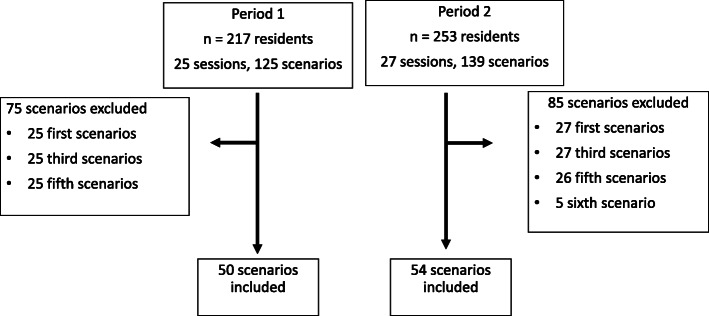
Flow chart for both periods of 1^st^ year residents’ simulation sessions about resuscitation in the delivery room: Step A

**Fig. 2:**
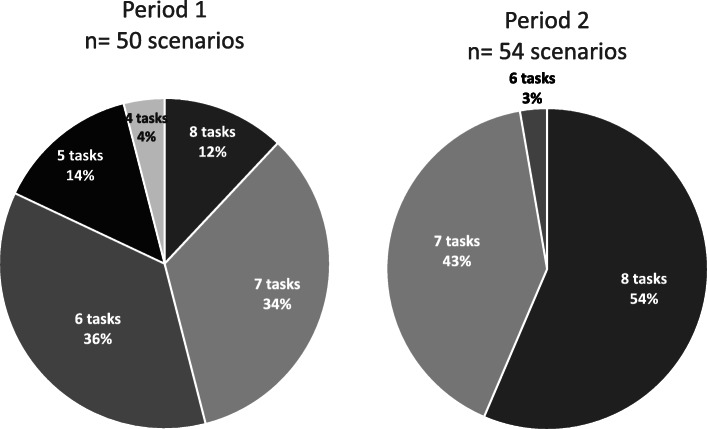
Number and percentage of tasks performed during neonatal resuscitation Step A (8 tasks required). Caption: Period 1 refers to the 2010 guidelines and period 2 to the 2015 guidelines

All first-year pediatric residents participated in the simulated newborn resuscitation sessions: 470 residents completed a total of 264 scenarios in 52 sessions (Fig. 1). Figure. 1 shows the flow chart of the study, Table 2, and Fig. 2, show the main results.

**Table 2:** Residents’ performances per period Positive Pressure Ventilation (PPV) initiation according to the European guidelines. Caption: Period 1: PPV initiation before 30 seconds according to the 2010 guidelines. Period 2: PPV initiation before 60 seconds according to the 2015 guidelines

	Period 1 (goal to PPV ≤ 30 sec) 50 scenarios	Period 2 (goal to PPV ≤ 60 sec) 54 scenarios	p
Time of PPV initiation (sec) Mean ± SD	63.9 + 15	59 + 14	NS
Number of tasks performed before PPV initiation per scenario Median [IQR]	6 [6-7]	8 [7-8]	*p* < 0.0001
Number of scenarios with 8 tasks completed before PPV initiation n (%)	6 (12%)	29 (54%)	*p* < 0.0001

The duration of scenarios and debriefings were the same in both periods.

In period 1, none of the pairs of learners was able to perform step A tasks and began PPV within 30 seconds as recommended in the 2010 guidelines.

PPV started at the same time in both periods (64 sec in period 1 vs. 60 sec in period 2, NS)

(Table 2), although the completion of the eight tasks of step A was significantly better during the second period (6 [6-7] vs. 8 [7-8] *p <* 0.001) (Table 2). The improvement in number and percentage of tasks completed for step A was significant in period 2 compared to period 1 (Fig. 2). Finally, we observed a significantly increased number of scenarios with total completion of step A before PPV start, during the second period (54% vs. 12%, *p <* 0.0001).

## Discussion

Despite the shorter allocated length in the first period, PPV start time did not differ between the two periods and roughly occurred 60 seconds after the start of resuscitation. Within the same time frame, however, task performance before PPV start was better in period 2 than in period 1. It should be remembered that between the two periods, the only change was the allocated time for PPV start according to the guidelines in use at that time: 30 seconds in period 1 and 60 seconds in period 2.

We explored a possible effect of time pressure on the trainees’ situation awareness. If we hypothesize that the core problem could be the time constraint placed on a task making people feel “time pressured” [11], it raises the question of the appropriate time determination for task execution. In period 1 none of the residents was able to perform step A as mandated by the 2010 guidelines [1]. None of them completed the eight tasks and began the ventilation before the first 30 seconds of resuscitation. It is notable that the 2015 guidelines [2] suggest the 30 second time for completing step A tasks was probably unreasonable. Perlman, Wyllie, Katwinkle et al, further assert in their consensus statement that this 30-second rule was not evidenced-based [12]. The crucial point requiring determination is the latest physiologic limit before PPV start without clinical consequences. There is some uncertainty about this time limit, but a comprehensive study in 2012 showed that about 93% of living newborns initiated spontaneous breathing in less than 30 seconds and 99% in less than 60 seconds [13]. In the worksheet which precedes the current Guidelines, we can only read that PPV should be done “as early as possible” [1]. The problem could be addressed in another way, i.e., by assessing if it is possible to follow the guidelines. This could be done in a multicenter simulation laboratory study involving experienced midwives, neonatologists, and pediatric intensivists and ask them to perform the various scenarios and measure the time to complete efficiently step A with acceptable time pressure. Video recording of real-world conditions could also be used to obtain this information [14, 15].

However, the time constraint is not only linked to the available time but also the number of cognitive events or cognitive load. An observational study in real life [14] showed that heart rate assessment, which is the last task of step A, needed to be done, was achieved in only 27% of the cases by a team receiving regular training. This study took place with premature newborns, but the tasks to be completed were the same, except “drying” replaced by “wrap in a bag.” There were no changes between the two periods in our study of the tasks performed while step A duration increased. So we could consider the time constraint was reduced by increasing the available time, whereas requiring time for task execution remained steady. According to Benson et al [5], this increase might lead to the reduction of time pressure; consequently, better execution of the requested tasks within the same time (significant increase of the number of executed tasks and of the number of dyad of residents able to perform all the tasks within 1 mn in the second period versus the first period).

Training might be a solution to decrease time pressure when facing time constraint, but some experiments reported the reverse. For instance, Zakay [16] found that under time pressure, training did not improve the quality of decision making. Similarly, Gonzalez et al [17] showed that despite additional practice runs, participants performed worse under high time constraint than did those working under a low time constraint. Although these studies were designed to evaluate the effect of time pressure on decision making and not on task execution, we can reasonably consider a relationship between the decision and the execution of tasks and could wonder whether these findings can also apply to the execution of tasks.

Adaptative strategies could also be a solution when facing time pressure. Studying the choice of adaptive strategies (i.e. work faster and do an imperfect job or work quicker and complete only part of the tasks) [4] adopted by time pressured people would be interesting. Besides, understanding the reasons why a given choice has been made remains unknown [11]. In our study, in the two periods, the residents could have been facing the following option: expedite the process of step A by forgetting some tasks to start step B at the recommended time, or decide to break the rule and voluntarily take more time before beginning step B [17]. In our learning sessions, when residents performed an imperfect job, understanding their choices and their adaptative strategies is a mandatory objective of the debriefing, but without recorded debriefings, we cannot evaluate these points in our study. Recording the debriefings can thus be interesting for further studies.

### Limitations:

The duration of scenarios and debriefings during the two periods were the same, but unfortunately, we did not record the debriefings. A point to consider is a possible improvement of the debriefings related to an increased experience of the debriefers. Even if the debriefing team always contained at least one novice, we cannot exclude that debriefing skills improved as sessions progressed and could affect the participants’ learning. However, the structure and the critical points of debriefing were predefined for each scenario and did not change during the study period.

Although we collected results for 6 years, our study was unicentric, and we included only first-year pediatric residents, leaving us a doubt as to how would a more experienced sample of physicians deal with the change of Phase A duration. However, we tried to minimize these biases with a high level of standardization, including a large number of residents providing a significant basis for analysis. Finally, without recorded debriefings and learner surveys, we are not able to assess adaptative strategies and their relationship to time pressure, according to different levels of time constraints.

We could not rule out the possibility of additional non-random training before our session but as they are first year residents they did not have any official training before the simulation session.

## Conclusions

When the 2015 guidelines doubled the time limit, a significant improvement in the completion of step A was noticed and was not associated with a delayed PPV start time. The 30-second - time constraint with step A as imposed by the 2010 European guidelines on neonatal resuscitation was associated with less than optimal performance of 1st-year pediatric residents.

This example suggests that guidelines that set a difficult-to-reach time threshold should consider not only the positive clinical effect on outcomes of a rapidly performed action but also the feasibility of the task associated with an important time constraint. Simulation-based training could be a way for testing the feasibility of guidelines, especially for time constraints.

We might suggest that reduced time pressure associated with the decreased time constraint could explain this improvement. Unfortunately, our study was not designed to answer this question, but it could be an interesting topic to be explored in future studies.

## Supplementary information

**Additional file 1.**

**Additional file 2.**

**Additional file 3.**

## Data Availability

The datasets used and/or analysed during the current study are available from the corresponding author on reasonable request.

## Abbreviations

HR: Heart rate
PPV: Positive pressure ventilation
