# Supplementary material for: Allowing more time to ILCOR Step A of neonatal resuscitation leads to better residents’ task completion in simulated scenarios. A problem of time pressure?
Source: BMC Pediatr. 2020 Jul 3;20:331. doi: 10.1186/s12887-020-02217-3 (PMC7333394; doi:10.1186/s12887-020-02217-3)
Supplement: Supplementary file 1 — Additional file 1. [file 12887_2020_2217_MOESM1_ESM.pdf]

Sessions structure for 1st year pediatric residents

Number	Scenarios	Learning objectives	Goals to achieve
1	Assessment of the newborn<br>Step A	Objective 1: Initiate resuscitation with correct Phase A<br>Objective 2: Recognition of a newborn not requiring PPV	-Realize complete phase A in less than 60 seconds
2	Initiating PPV<br>Step B	**Objective 1: Initiate resuscitation with correct Phase A**<br>Objective 2: Recognition of a newborn requiring PPV<br>Objective 3: Demonstrate correct technique for positive ventilation, including placement of mask on the newborn's face, rate and pressure, and corrective actions for ineffective PPV	**-Realize complete phase A in less than 60 seconds**<br>**-Initiate PPV before 60 seconds WITH a complete phase A before**
3	Initiating PPV in a context of meconial amniotic fluid<br>Step B	Objective 1: Initiate resuscitation with correct Phase A<br>Objective 2: Recognition of a newborn requiring PPV<br>Objective 3: Apply recommendation of ventilation in a context of meconial amniotic fluid	-Realize complete phase A in less than 60 seconds<br>-Initiate PPV before 60 seconds with a complete phase A before<br>-Deny intubation
4	Initiating PPV and assess dependency to PPV<br>Step B	**Objective 1: Initiate resuscitation with correct Phase A**<br>Objective 2: Start PPV in the right time<br>Objective 3: Demonstrate correct technique for positive ventilation, including placement of mask on the newborn's face, rate and pressure, and corrective actions for ineffective PPV	**-Realize complete phase A in less than 60 seconds**<br>**-Initiate PPV before 60 seconds with a complete phase A before**<br>-Assess dependency to the PPV, discuss intubation
5	Initiating PPV and discussing Chest compression<br>Step B± Step C	Objective 1: Initiate resuscitation with correct Phase A<br>Objective 2: Start PPV in the right time<br>Objective 3: Assess the opportunity to start chest compressions	-Realize complete phase A in less than 60 seconds<br>-Initiate PPV before 60 seconds with a complete phase A before<br>-Don't start chest compressions

Caption: In bold characters, elements linked to the study
