# Supplementary material for: Allowing more time to ILCOR Step A of neonatal resuscitation leads to better residents’ task completion in simulated scenarios. A problem of time pressure?
Source: BMC Pediatr. 2020 Jul 3;20:331. doi: 10.1186/s12887-020-02217-3 (PMC7333394; doi:10.1186/s12887-020-02217-3)
Supplement: Supplementary file 2 — Additional file 2. [file 12887_2020_2217_MOESM2_ESM.pdf]

CELESTINE (OP2-1)

PHASE B
Sim new B

**Scenario Script Célestine**	
**Case Presentation (to be read to participants):**	You are called to attend a vaginal delivery of a 39-week infant because of difficult assisted delivery. The estimated weight is 3,000 grams (3 kg).<br>The mother is 30-year-old, G1 P1. The pregnancy was uncomplicated. The labour started spontaneously 12 hours ago. The amniotic fluid was clear.<br>The foetal heart rate was normal<br>The mother has been in the second stage of labour for 2 hours, and within the last 20 minutes, foetal heart rate tracings have become concerning due to intermittent late decelerations.<br>The obstetrical team decided an assisted delivery by vacuum extraction. The extraction is difficult. At birth, the infant is noted to have tight nuchal cord x 2 requiring surgical reduction.
**Facilitator 1 Midwife**	• **Bold Point in prebriefing →**<br>• Make feel it could be a stressfull situation<br>• Exit from the room, learners have to prepare what they could need<br>• **Role in the scenario**<br>○ Come with the newborn, tells she doesn't cry and she is floppy<br>○ Birthweight is heavier, more than 4000g<br>○ She has to leave because there is a second delivery in another delivery room<br>○ If learners stucked, suggests ventilation

**Pedagogic sheet**	
**Learning objectives**	Objective 1: Initiate resuscitation with correct Phase A<br>Objective 2: Recognition of a newborn requiring PPV
**Stage of the scenario Trigger:**	**Initial state**: RR 6/min, SpO_2 65%, HR 80/min, weak cry
**1-Ventilation**	**Ventilation:**<br>RR 40/min, SpO_2 80%, HR 130/min, increase HR after 20 seconds of ventilation
**2- If no action**	**No ventilation:** Trigger Time 1 mn 10" leads to bradycardia 65 stop weak cry.
**3 Scenario's ending**	RR 55/min, SpO_2 90%, HR 150/min at 5 minutes
**Key points of the debriefing**	• **PPV at the right time**<br>• **Communication between learners**<br>○ ILCOR Step A<br>○
