# Supplementary material for: Allowing more time to ILCOR Step A of neonatal resuscitation leads to better residents’ task completion in simulated scenarios. A problem of time pressure?
Source: BMC Pediatr. 2020 Jul 3;20:331. doi: 10.1186/s12887-020-02217-3 (PMC7333394; doi:10.1186/s12887-020-02217-3)
Supplement: Supplementary file 3 — Additional file 3. [file 12887_2020_2217_MOESM3_ESM.pdf]

LINDSAY (OP2-1)

PHASE B
Sim new B

**Scenario Script Lindsay**	
**Case Presentation (to be read to participants):**	You are called to attend a vaginal delivery of a 39-week infant because mother is psychiatric (schizophrenia). The mother is 26-year-old, G1 P1. Treatment was Haloperidol Olanzapine, Oxazepam. The pregnancy was uncomplicated, but follow up of the pregnancy began late at 20 weeks. The labour started spontaneously 6 hours ago. The amniotic fluid was clear. The foetal heart rate was normal.<br>The mother wants to leave to go back to the psychiatric hospital. Talk is incoherent<br>The anesthesiology team decided to perform sedation (no opioids). Quick birth after sedation
**Facilitator 1 Midwife**	• **Bold Point in prebriefing →**<br>• Make feel it could be an unusual situation.<br>• Exit from the room, learners have to prepare what they could need<br>• **Role in the scenario**<br>○ Come with the newborn, tells she doesn't cry and she is floppy<br>○ Birthweight is 3000g<br>○ She has to leave because there is a second delivery in another delivery room<br>○ If learners stucked, suggests ventilation

**Pedagogic sheet**	
**Learning objectives**	Objective 1: Initiate resuscitation with correct Phase A<br>Objective 2: Recognition of a newborn requiring PPV and dependant to the PPV<br>Objective 3: Discuss the potential need for intubation
**Stage of the scenario Trigger:**	**Initial state**: RR 5/min, SpO_2 65%, HR 80/min, grunting, limp
**1-Ventilation**	**Ventilation:**<br>RR 40/min, SpO_2 80%, HR 130/min, increase HR after 20 seconds of ventilation
**2- Stop Ventilation**	**No ventilation:** leads to bradycardia 80 stop weak cry, RR 0, SpO_2 74.
**3 Resuming ventilation**	**Resuming ventilation:**<br>RR 55/min, SpO_2 90%, HR 150/min at 5 minutes<br>End of the scenario
**Key points of the debriefing**	• **PPV at the right time**<br>• **Dependancy to PPV, what to do in this case**<br>• **Communication between learners**<br>○ ILCOR Step A<br>○
